# Therapeutic effect of TMZ-POH on human nasopharyngeal carcinoma depends on reactive oxygen species accumulation

**DOI:** 10.18632/oncotarget.6410

**Published:** 2015-11-27

**Authors:** Li Xie, Xingguo Song, Wei Guo, Xingwu Wang, Ling Wei, Yang Li, Liyan Lv, Weijun Wang, Thomas C. Chen, Xianrang Song

**Affiliations:** ^1^ Shandong Provincial Key Laboratory of Radiation Oncology, Shandong Cancer Hospital and Institute, Jinan, Shandong Province, China; ^2^ Ultrasound Diagnosis Department, Shandong Cancer Hospital and Institute, Jinan, Shandong Province, China; ^3^ Department of Neurological Surgery and Pathology, University of Southern California, Los Angeles, CA, United States

**Keywords:** nasopharyngeal carcinoma (NPC), reactive oxygen species (ROS), temozolomide (TMZ), perillyl alcohol (POH)

## Abstract

Nasopharyngeal carcinoma (NPC) is a common head and neck malignancy without efficient chemotherapeutic agents for it. In our current study, we demonstrated the cytotoxicity effects of a newly patented compound temozolomide–perillyl alcohol (TMZ-POH) on NPC *in vitro* and *in vivo*, and the possible mechanisms involved. Human NPC cell lines CNE1, CNE2, HNE2, and SUME-α were treated with control (DMSO), TMZ, POH, TMZ plus POH, and TMZ-POH. Our data indicated that TMZ-POH could inhibit NPC cell proliferation, cause G_2_/M arrest and DNA damage. TMZ-POH triggered apoptosis in NPC cells via significant activation of caspase-3 and poly(ADP-ribose) polymerase (PARP). Importantly, TMZ-POH-induced cell death was found to be associated with (i) the loss of inner mitochondrial membrane potential (ΔΨm) and release of mitochondrial Cytochrome *c*, (ii) the increase in ROS generation, and (iii) the activation of stress-activated protein kinases (SAPK)/*c*-Jun N-terminal kinases (JNK) signaling pathway. The generation of ROS in response to TMZ-POH seems to play a crucial role in the cell death process since the blockage of ROS production using the antioxidant *N*-acetyl-L-cysteine or catalase reversed the TMZ-POH-induced JNK activation, DNA damage, and cancer cell apoptosis. These results provide the rationale for further research and preclinical investigation of the antitumor effect of TMZ-POH against human NPC.

## INTRODUCTION

Nasopharyngeal carcinoma (NPC) is a common head and neck malignancy with distinct ethnic and geographic distribution. It is endemic in southern China and Southeast Asia. A potential link exists between Epstein–Barr virus and the development of NPC [1, 2]. Given its high radiosensitivity, the standard treatment for NPC is radiotherapy. Along with the damage of radiation to normal tissues, radioresistance remains a serious obstacle to successful treatment. Radioresistance can cause local recurrence and distant metastases in some patients [3]. Conventionally, chemotherapy is given concurrently with radiotherapy for treating locally advanced disease. However, the overall survival after recurrence is usually poor with reported median survival ranging from 7.2 to 22 months [4, 5]. Thus, special emphasis is on the discovery of effective chemotherapeutic agent.

Temozolomide (TMZ) is a DNA alkylating agent that is currently the standard care medication administered to glioblastoma multiforme patients as its transient tumor growth–arrest property [6, 7]. The therapeutic benefit of TMZ depends on its ability to alkylate DNA at the *N*^7^ or *O*^6^ position of guanine residues; high expression levels of the cellular repair enzyme *O*^6^-methylguanin-DNA-methltransferase (MGMT) can protect tumor cells from the cytotoxic impact of TMZ, which has been demonstrated the contribute to treatment resistance [8–10]. Perillyl alcohol (POH) is a naturally occurring monoterpene that is used orally for treating a variety of cancers, including breast, pancreas, and lung carcinomas [11–13], and has an amazing capability to enhance the cytotoxicity of TMZ in several tumors, including the TMZ-resistant gliomas [14]. In this study, POH was covalently conjugated to TMZ, thereby generating a novel TMZ analog TMZ-POH (honorable product from NeOnc Technologies), which displayed a greater anticancer potency than each of its parental molecules in several types of malignant neoplasms such as TMZ-resistant gliomas, triple-negative breast cancer [15], and melanoma [16].

TMZ-POH is lipid soluble, so it can be used via inhalation or nasal drip. Direct deposition of TMZ-POH at the nasopharyngeal site can increase nasopharyngeal local drug concentrations; reduce the overall dose required, thereby reducing the side effects that result from high doses; and also lead to increased patient convenience [17]. Based on these promising results, in this article, the anticancer activity of TMZ-POH was evaluated against NPC in comparison with TMZ, POH, or a mix of TMZ plus POH *in vitro* and *in vivo*.

It is well established that reactive oxygen species (ROS) play important biological roles in cell homeostasis. Several studies have also reported that high intracellular ROS levels are usually associated with apoptosis in cancer cells [18]. The well-characterized mitogen-activated protein kinase (MAPK) family member, stress-activated protein kinases/*c*-Jun N-terminal kinase (SAPK/JNK), plays important roles in the coordination of cellular stress responses toward different stimuli including ROS [19], and is an important mediator of apoptosis induction in response to different chemotherapeutic agents. The results of this study provide a novel mechanism of TMZ-POH that ROS accumulation is involved in TMZ-POH-induced JNK activation, DNA damage, and cancer cell apoptosis.

## RESULTS

### Cytotoxicity of TMZ-POH on the growth of NPC cells *in vitro*

**Figure 1:**
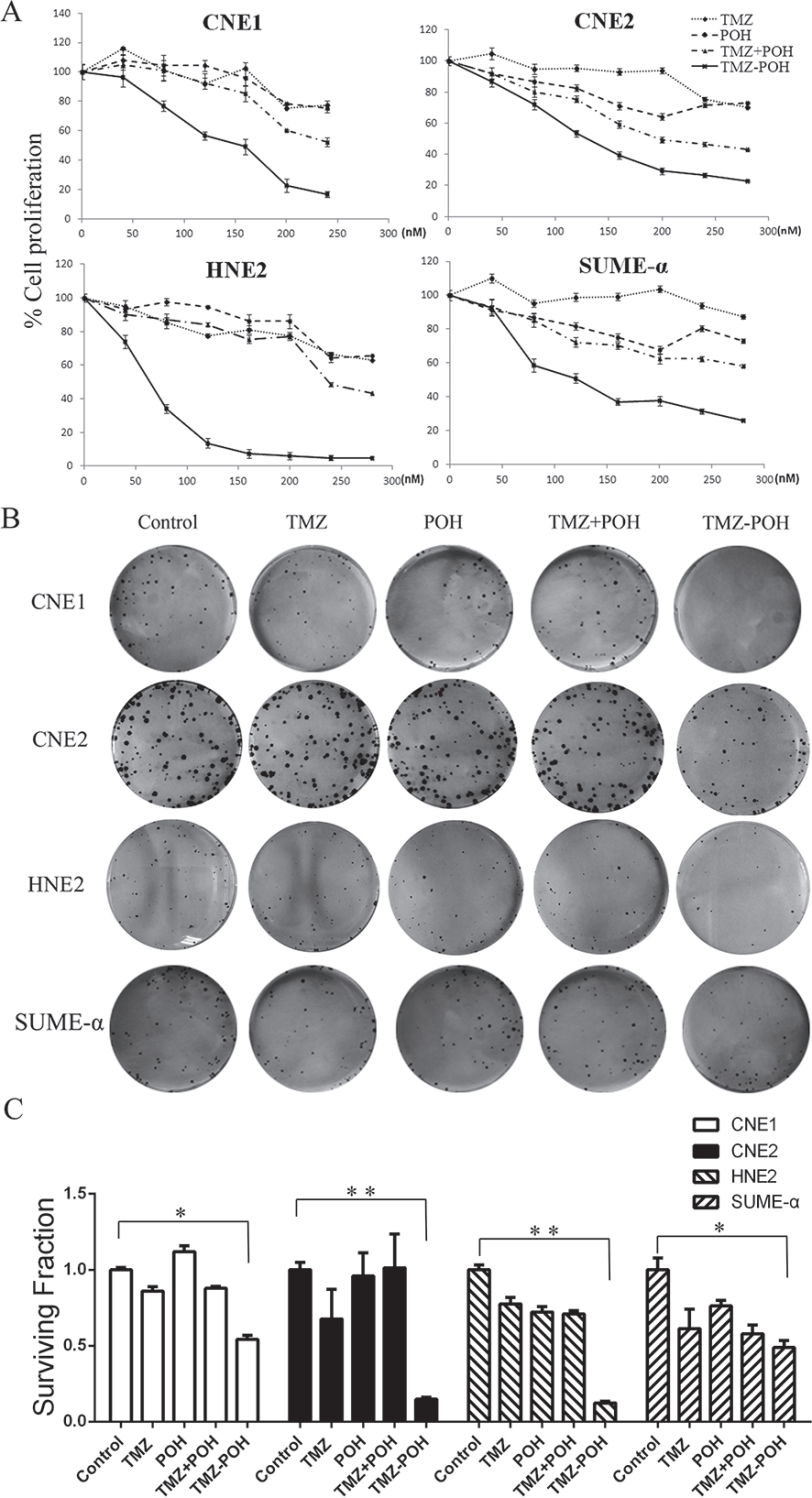
Effects of TMZ-POH on the growth of NPC cells (**A**) CNE1, CNE2, HNE2, and SUME-α cells were treated with the indicated concentrations of TMZ, POH, TMZ plus POH, and TMZ-POH for 48 hours, and then subjected to the MTT assay. The absorbance value was calculated and standardized to the control (DMSO) group. (**B**) The above cells were treated with 100 μM TMZ, POH, TMZ plus POH, and TMZ-POH for 2 hours and subjected to the cell colony formation assay. (**C**) Surviving fraction is presented as mean ± SD, *P < 0.05, **P < 0.01 (*n* = 3 in each group).

To verify the toxic role of TMZ-POH in NPC, four NPC cell lines CNE1, CNE2, HNE2, and SUME-α were employed in our study, which were treated with several concentrations of the individual constituents (TMZ-POH, TMZ, or POH) alone, or with an equimolar mix of TMZ plus POH for 48 hours, and the cell viability was determined by MTT assay. As shown in Figure 1A, TMZ-POH inhibited the proliferation of CNE1, CNE2, HNE2, and SUME-α compared to its individual constituents (TMZ, POH) and their combination (TMZ plus POH) in a dose-dependent manner significantly. Furthermore, a colony formation assay was also carried out, which demonstrated a more potent inhibition of colony formation capability triggered by TMZ-POH than by its constituents and their combination (Figure 1B and Figure 1C). Taken together, the data in this study supported the inhibitory role in NPC cell growth and colony formation.

### Cytotoxicity of TMZ-POH on the growth of NPC cells *in vivo*

**Figure 2:**
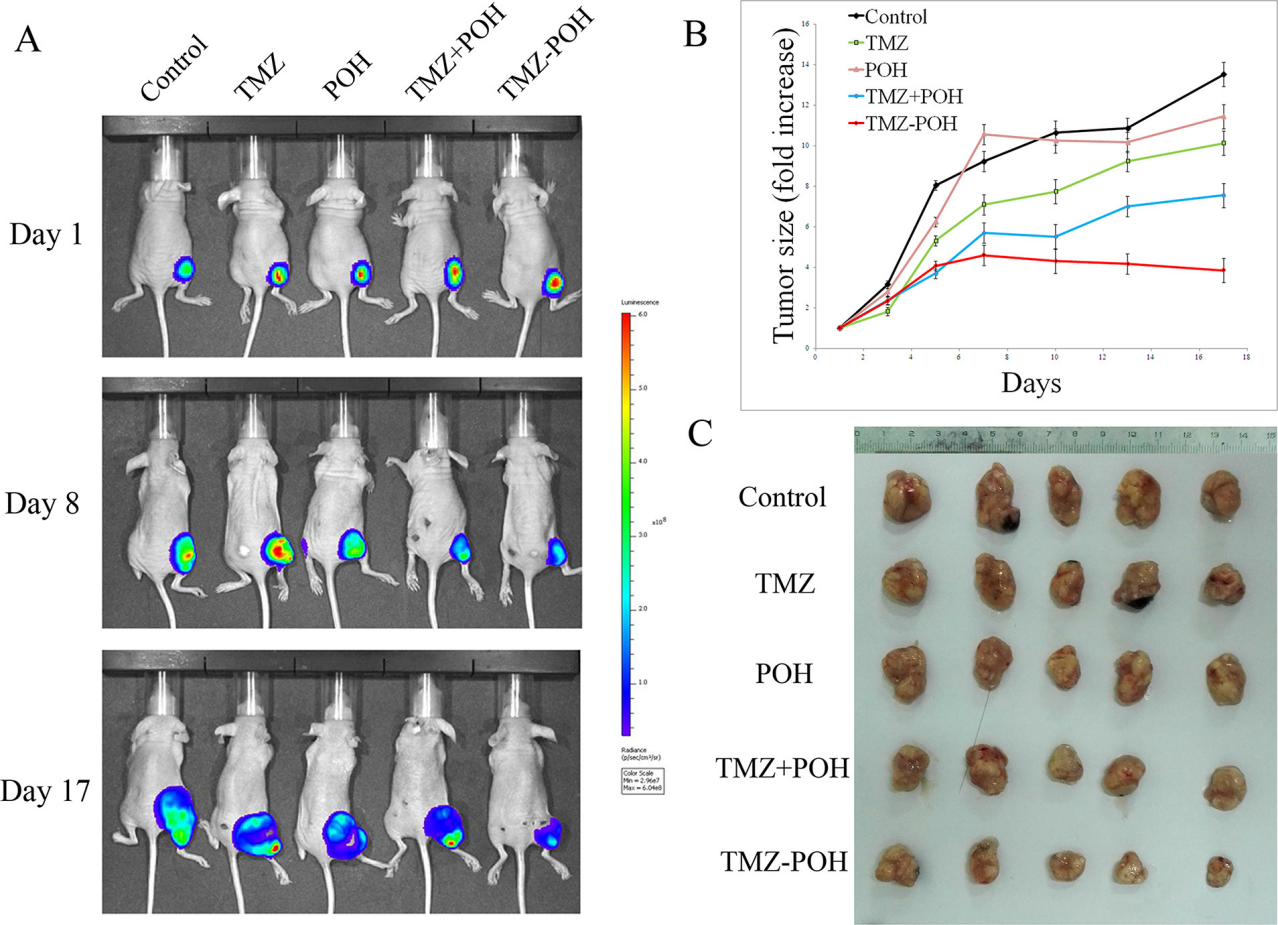
Effect of TMZ-POH on subcutaneous tumor growth (**A**) Mice carrying subcutaneously implanted HNE2/luc cells were separated into five treatment groups (five animals each): (i) control (DMSO only), (ii) 50-mg/kg TMZ, (iii) 50-mg/kg POH, (iv) 22-mg/kg POH mixed with 28-mg/kg TMZ (mimicking the dosage of the individual components contained in 50-mg/kg TMZ-POH), and (v) 50-mg/kg TMZ-POH, all injected subcutaneously. Representative images of treated tumors with different treatment were detected. (**B**) Tumor size was calculated in these animals after the cessation of treatment. Both graphs show folds increase in tumor size over time. (**C**) Image of HNE2/luc primary tumor in mice 17 days after the cessation of treatment in different treatment groups.

To evaluate the *in vivo* anticancer activity of TMZ-POH, the growth inhibition of HNE2 xenografts in nude mice was investigated. All animals were imaged for luciferase expression to confirm efficient tumor uptake. Figure 2 presented tumor growth in these animals after the cessation of treatment. As shown in Figure 2B, all control animals exhibited much increased bioluminescent radiance (indicative of vigorous tumor growth); the administration of TMZ-POH resulted in the significant growth suppression of HNE2 xenografts when compared with the control groups (*p* < 0.05). Tumor growth showed less bioluminescence in the TMZ-POH-treated group than in other groups, indicating that the therapeutic efficacy of TMZ-POH was substantially stronger than that of TMZ, POH, or their combination. No statistically significant differences in the body weights were observed between the mice in TMZ-POH-treated group and mice in control group (*P* > 0.05, data not shown), indicating a low general toxicity of TMZ-POH. These data show that TMZ-POH exhibits potent antitumor activity and high safety *in vivo*.

### Role of TMZ-POH treatment in G_2_/M arrest and DNA damage repair pathway activation

**Figure 3:**
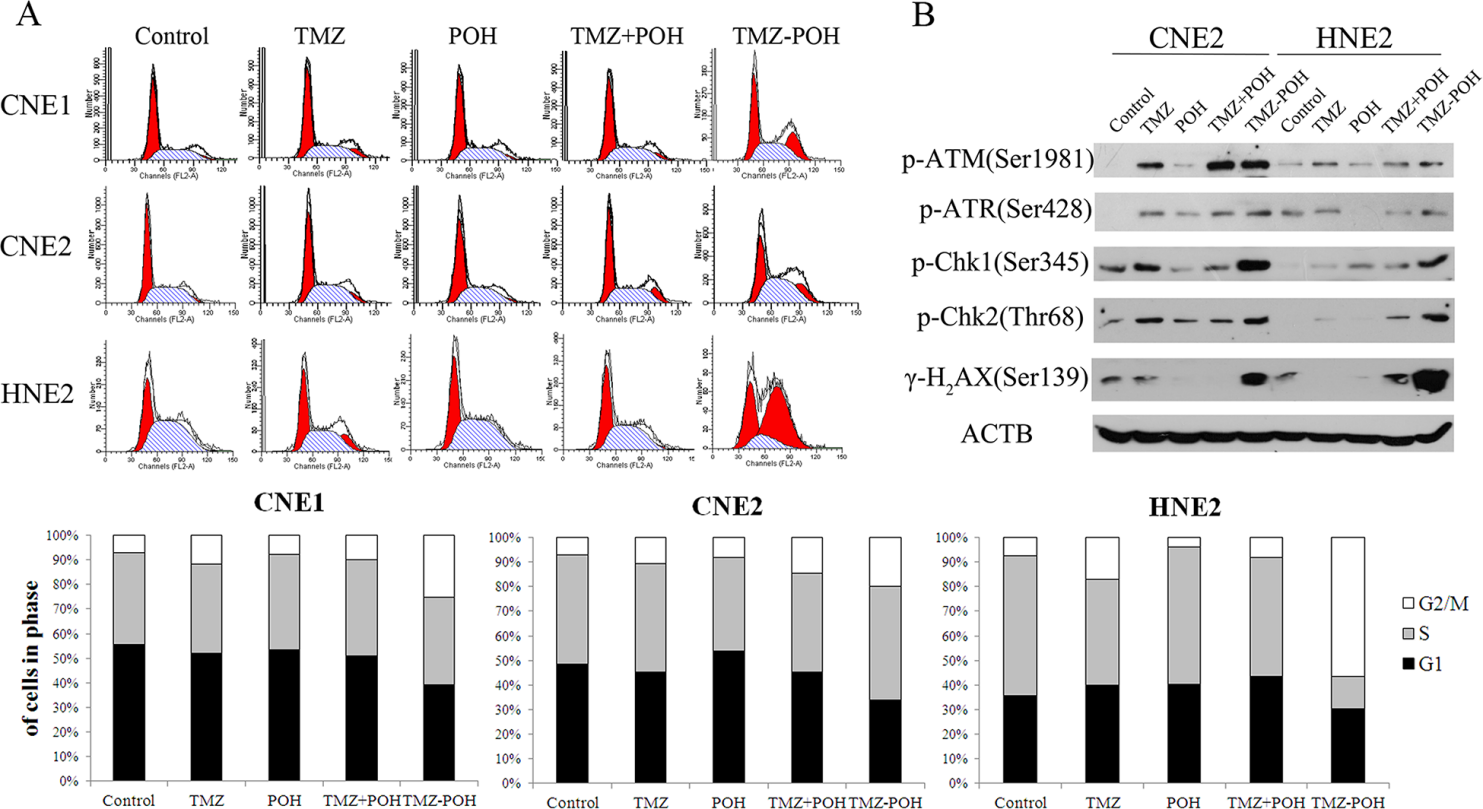
Effects of TMZ-POH on cell cycle checkpoint/DNA repair pathway in NPC cells (**A**) The cell cycle distributions of CNE1, CNE2, and HNE2 were analyzed by flow cytometry after 36 hours of treatment with the indicated constituents (100 μM); representative images are shown. Three independent experiments were performed. (**B**) DNA repair pathway was detected by Western blotting after 24 hours of treatment with the indicated constituents (100 μM).

TMZ is shown to exert its cytotoxicity by inducing DNA double-strand breaks (DSBs). Therefore, whether TMZ-POH also induced cell death via DNA damage was investigated. In previous studies, DNA damage was reported to result in cell cycle arrest and induce a DNA damage repair response [20–22]. To address this issue, cell cycle and DNA damage repair pathway were studied. As shown in Figure 3A, TMZ-POH led to an obvious G_2_/M arrest compared to its individual constituents and their combination. ATM (ataxia telangiectasia mutated) and ATR (ataxia telangiectasia and Rad3-related) kinases are generally activated in response to DNA damage [23, 24]. Indeed, TMZ-POH was observed to induce the phosphorylation of ATM and ATR at the Ser1981 and Ser428 site, respectively (Figures 3B and 5D). Chk1 and Chk2 are the cell cycle checkpoint kinases, downstream of ATM and ATR [23, 25], and were shown to be phosphorylated at the Ser345 and Thr68 site, respectively (Figures 3B and 5D). When DNA is damaged, DSBs trigger the recruitment of ATM to the damaged site, which in turn phosphorylates histone H_2_AX (yielding γ-H_2_AX) resulting in foci formation at the damage side [26]. As shown in Figures 3B and 5D, TMZ-POH treatment induced an increase in phosphorylated H_2_AX (Ser139). Together, these results characterize TMZ-POH as an alkylating agent with cytotoxic mechanism similar to TMZ, but with a potency that is substantially greater than the original compound.

### TMZ-POH-induced apoptosis in human NPC cells

**Figure 4:**
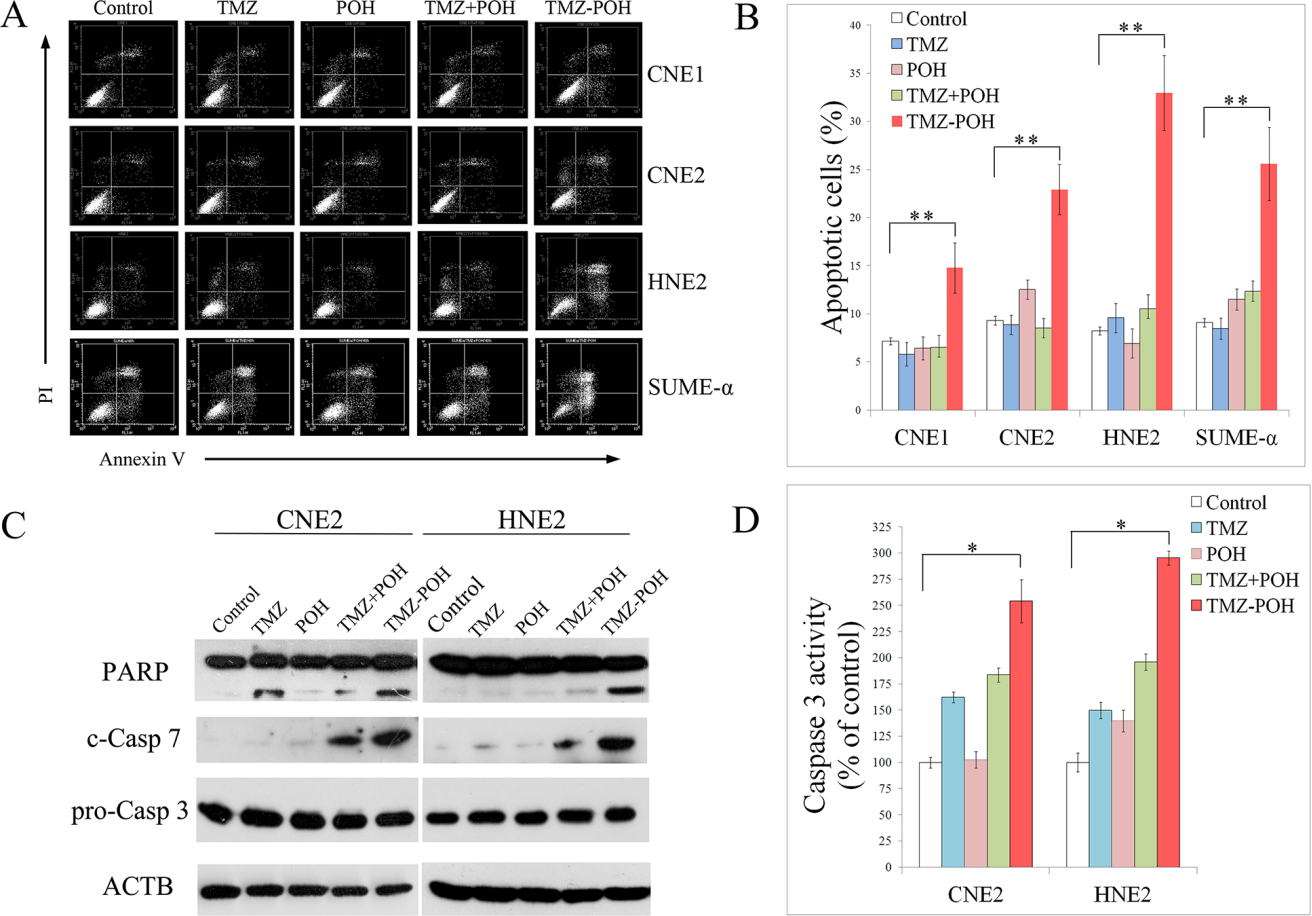
Effect of TMZ-POH on the apoptosis of NPC cells (**A**) Apoptosis was analyzed by Annexin V/PI staining after 24 hours of treatment with the indicated constituents (100 μM). (**B**) Representative histograms are shown. (**C**) The expression of apoptosis-related proteins was detected by Western blotting after 24 hours of treatment with the indicated constituents (100 μM). (**D**) Activation of caspase-3 was determined by ELISA. The results shown are means ± SD; *P < 0.05, **P < 0.01.

**Figure 5:**
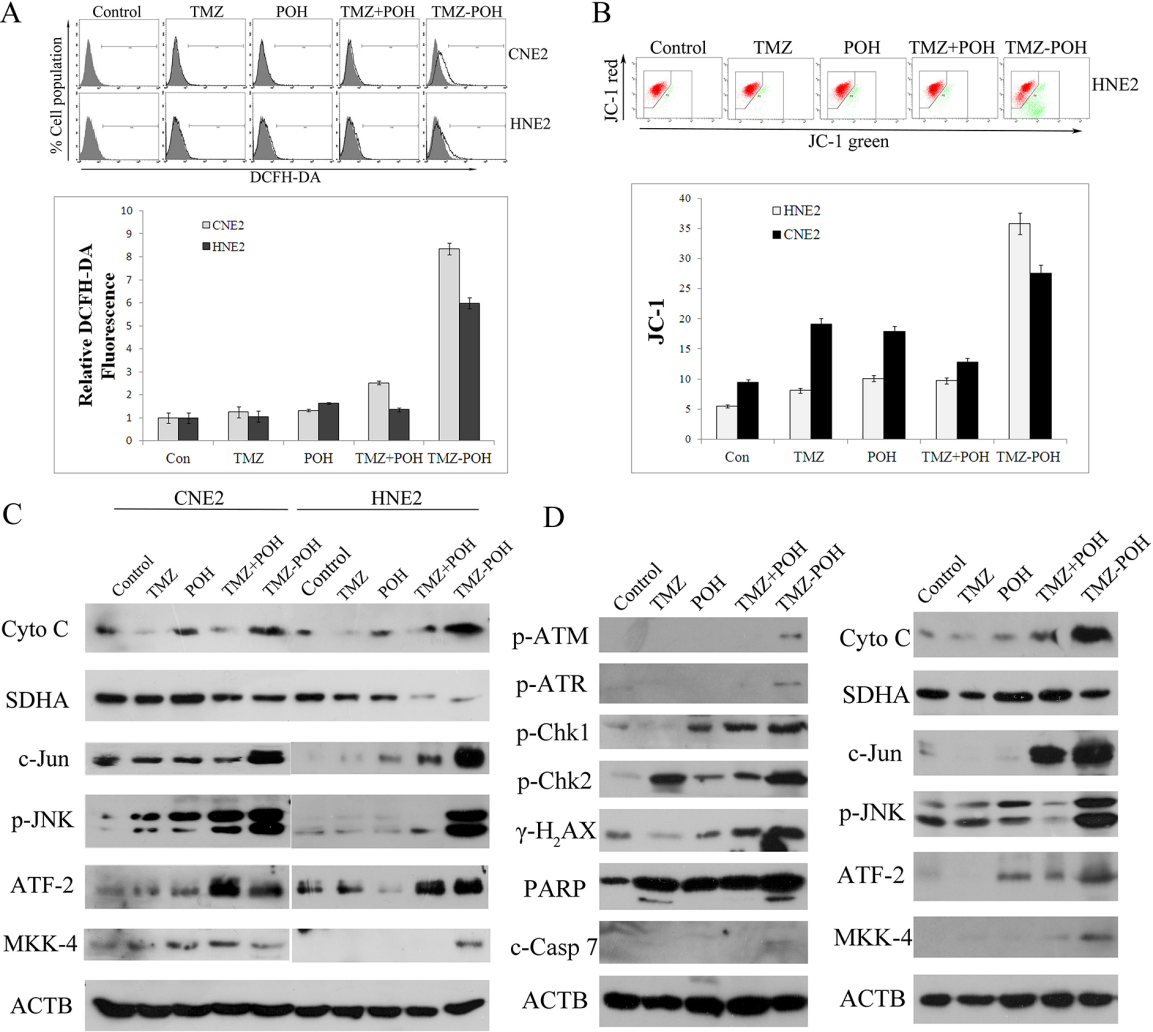
TMZ-POH-induced ROS accumulation, MTP decrease, and activation of JNK–mitochondrial apoptotic pathways (**A**) Intracellular ROS levels were measured using the fluorescent probe DCFH-DA after cells were treated with the indicated constituents (100 μM) for 24 hours. The raw data from each individual experiment were normalized to control cells. (**B**) MMP assays by flow cytometry using the JC-1 kit. The results shown are means ± SD. (**C**) Western blot analysis of Cyto *c*, SDHA, *c*-Jun, p-JNK, ATF-2, and MKK-4 in CNE2 and HNE2 cells treated with the indicated constituents (100 μM) for 24 hours. (**D**) The expression of proteins in tumor specimens in mice was detected by Western blotting 3 days after the cessation of treatment in different treatment groups (Figure 2A). ACTB was used as internal control. Data presented are representative of three independent experiments.

Next, whether TMZ-POH can induce NPC cell apoptosis was investigated. As shown in Figure 4A and 4B, TMZ-POH treatment resulted in a significant increase in the percentage of Annexin V–positive cells in all above cells compared to other constituents. Next, whether the superior effect of TMZ-POH would also be reflected at the molecular level of apoptosis-related protein expression was determined. Western blot analysis showed that TMZ-POH increased significantly the expression of cleaved PARP and cleaved (i.e., activated) caspase-7 in CNE2 and HNE2 cells (Figure 4C). Caspase-3 is a key effector in the process of apoptotic cell death. As shown in Figure 4D, the elevation of activated caspase-3 was found remarkably profound in TMZ-POH-treated group in both HNE2 and CNE2 cells. All three indicator proteins were induced quite prominently by TMZ-POH after 1 days of treatment, whereas TMZ, POH, or TMZ plus POH exerted noticeably weaker effects. Thus, the results from the cell survival assay (Figure 1A) correlated closely with the effects of these compounds on DNA damage and apoptosis markers (Figures 3B, 4C and 5D), and in all cases TMZ-POH clearly generated the strongest anticancer impact.

### Role of TMZ-POH in ROS accumulation and MTP decrease

ROS plays an important role in tumorigenesis and chemotherapy of most anticancer drugs. To assess the role of ROS in the anticancer effect of TMZ-POH in NPC cells, intracellular ROS levels were measured using DCFH-DA. As shown in Figure 5A, a significant sixfold to eightfold increase in ROS production was observed in both CNE2 and HNE2 cells upon TMZ-POH treatment, indicated by an increased DCFH-DA fluorescence intensity, suggesting the intracellular ROS levels were enhanced after the TMZ-POH treatment.

The mitochondrion is a major site of ROS generation in mammalian cells, the decreased mitochondrial membrane potential (MMP) is also a marker of apoptosis. So, MMP collapse was also examined. As expected, TMZ-POH led to a decrease in mitochondrial membrane potential (ΔΨm) significantly compared to other constituents (Figure 5B), as indicated by increased JC–1 fluorescence signal ratio (the fluorescent intensity of green to red). Coincidently, the release of Cytochrome *c* as the prototypic event for the induction of mitochondrial changes during apoptosis was induced by TMZ-POH, and the complements of antioxidant enzymes that modulate cellular ROS flux, such as SDHA1, were significantly suppressed by the TMZ-POH treatment.

For many drugs, the ability to induce apoptosis and cell cycle alterations in target cells is related to the signaling by JNKs, a family of serine/threonine kinases that mediate intracellular signal transduction in response to different physiological stimuli and stressing conditions. Therefore, the role of JNKs in cell cycle regulation and apoptosis induced by TMZ-POH in NPC cells were examined in the current study. As shown in Figure 5C and 5D, exposure of CNE2 and HNE2 cells to TMZ-POH induced phosphorylation of JNK, *c*-Jun, activating transcription factor 2 (ATF2), and mitogen-activated protein kinase kinase 4 (MKK4). Collectively, the data in this study suggest TMZ-POH leads to an imbalance of the cellular redox potential and ultimately programmed cell death.

### ROS-mediated cytotoxicity of TMZ-POH on NPC cells

**Figure 6:**
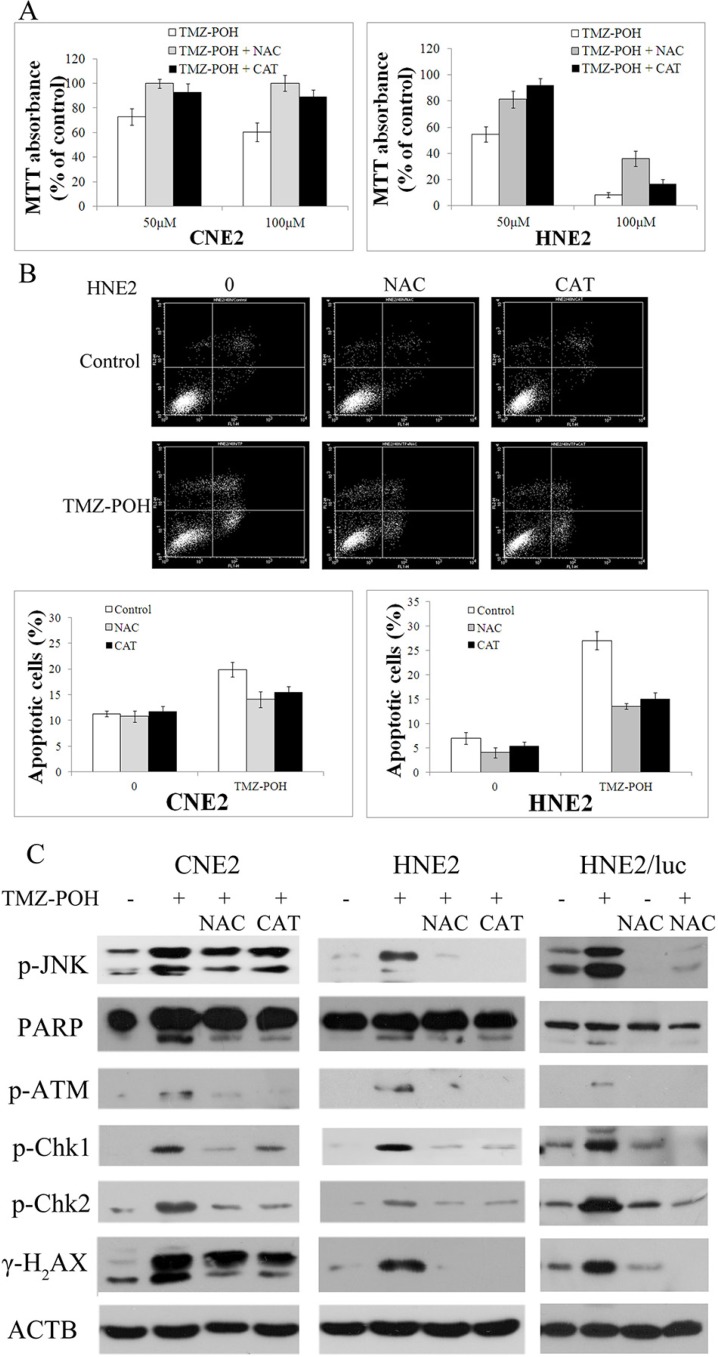
TMZ-POH-induced cytotoxicity is dependent on intracellular ROS generation CNE2 and HNE2 cells were preincubated with or without CAT or NAC for 2 hours before exposure to TMZ-POH (100 μM) for 48 hours. (**A**) The cell viability was determined by the MTT assay. (**B**) Percentage of cell apoptosis was determined by Annexin V/PI staining and flow cytometry. The results shown are means ± SD. (**C**) Mice carrying subcutaneously implanted HNE2/luc cells were separated into four treatment groups (five animals each): (i) control (DMSO only), (ii) 50-mg/kg TMZ-POH, (iii) DMSO and 100-mg/kg NAC, and (iv) 50-mg/kg TMZ-POH and 100-mg/kg NAC, NAC was injected intraperitoneally. Tumor specimens in mice was detected by Western blotting 3 days after the cessation of treatment. Cell lysates from the above treated cells (CNE2, HNE2) and tumor specimens in mice (HNE2/luc) were subjected to Western blot to analyze the expression of JNK pathway, DNA damage, and apoptosis-related factors. ACTB was used as an internal control. Data presented are representative of three independent experiments.

Next, as increased ROS production is critical in inducing cell apoptosis, whether TMZ-POH induced death is ROS dependent was determined. Two ROS scavengers, catalase (CAT) N-acetyl-L-cysteine (NAC) were employed to prevent ROS accumulation. The cell viability in TMZ-POH-treated HNE2 and CNE2 cells was restored in the presence of CAT or NAC (Figure 6A). The increase of apoptotic cells observed upon treatment with TMZ-POH was significantly reduced when cells were co-treated with CAT or NAC (Figure 6B). This ability of ROS inhibitor to abolish TMZ-POH-induced cytotoxicity suggested that TMZ-POH-induced cell death is ROS mediated.

Accumulating evidence support the crucial role of oxidative DNA damage. To confirm the relationship between ROS accumulation and DNA damage, the DNA damage–related protein expression after CAT or NAC treatments was detected *in vitro* and *in vivo*. As shown in Figure 6C, CAT or NAC efficiently inhibited the increase of cleaved PARP, p-ATM, p-Chk1, p-Chk2, and γ-H_2_AX induced by TMZ-POH, indicating TMZ-POH exhibits its cytotoxicity via oxidative DNA damage. No changes in HNE2 and CNE2 control cells were observed after the CAT or NAC treatment alone (data not shown). Also, the increase in JNK phosphorylation observed in TMZ-POH-treated HNE2 and CNE2 cells was in a ROS-dependent manner (Figure 6C).

This study demonstrates that TMZ-POH-induced cell death is largely regulated by a ROS-mediated DNA damage mechanism. It further demonstrates that TMZ-POH inhibits cell cycle progression and induces apoptosis in NPC cells by a ROS-mediated DNA damage mechanism (Figure 6).

## DISCUSSION

The current study provides a novel report on the cytotoxic effect of TMZ-POH on several different NPC cell lines *in vitro* and *in vivo* and the possible mechanisms. It was then observed that TMZ-POH significantly induced cell cycle arrest and cell apoptosis in all the four NPC cell lines (Figure 1). Besides the cellular effects, TMZ-POH was shown to be highly effective on inhibiting tumor growth in a tumor model using nude mice (Figure 2).

It was reported that TMZ could methylate DNA directly and activate ATM, which in turn transmits the DNA damage signal to downstream substrates, such as phosphorylated Chk1 and Chk2. The latter were well established to be key mediators for cell cycle. Therefore, DNA damage under TMZ-POH treatment was detected. TMZ-POH led to an obvious G_2_/M arrest and activation of the signaling checkpoints in response to DNA damage in NPC cell lines.

The newly designed compound TMZ-POH displayed greater anticancer potency than each of its parental molecules in NPC cells, as evident from a stronger inhibition of cell/tumor proliferation and colony formation, along with the higher level of G_2_/M arrest, apoptosis, and DNA damage. Similarly, TMZ-POH displayed a greater therapeutic efficacy than TMZ or POH *in vivo*. Intriguingly, a mere mix of the constituents of TMZ-POH, TMZ and POH, was unable to achieve the superior efficacy of TMZ-POH, neither *in vitro* (Figure 1 and Figure 4) nor *in vivo* (Figure 2), indicating that TMZ-POH is a novel chemical entity with inherently increased potency that is greater than the sum of its parts.

To elucidate the unique mechanism underlying the superior activity of TMZ-POH compared with TMZ or TMZ plus POH, potentially relevant signaling pathways were investigated. Many investigators have demonstrated that mitochondria are key regulators of apoptosis [27]. Apoptosis stimuli can cause MMP loss and Cytochrome *c* release from the mitochondria to the cytosol. In the cytosol, Cytochrome *c* activates caspase-3, after which specific substrates of caspase-3, such as PARP, are cleaved. PARP is a downstream substrate of activated caspases and protects DNA against oxidative damage. This process eventually leads to apoptosis [27, 28]. The present study indicates that the decrease of MMP, release of Cytochrome *c*, and repression of mitochondrial enzymes SDHA as the prototypic event for inducing mitochondrial changes during apoptosis are induced by TMZ-POH.

As ROS are generated mainly as byproducts of mitochondrial respiration, mitochondria are thought to be the primary target of oxidative damage [29]. The Overproduction of intracellular ROS may attack cellular membrane lipids, proteins, and DNA, and cause oxidative injury, and finally result in the reduction of cell cycle arrest or/and cell apoptosis to repair or eliminate the damaged cells [30–32]. Then, ROS levels in TMZ-POH-treated cells were also determined. TMZ-POH could induce ROS production in both CNE2 and HNE2 cells (Figure 5). More importantly, the ability of TMZ-POH to induce cytotoxicity was abrogated in the presence of two ROS scavengers, CAT or NAC. After efficiently preventing ROS accumulation, CAT and NAC treatments significantly alleviated the cytotoxicity of TMZ-POH, as revealed by the restoration of cell viability (Figure 6A), along with a remarkable decrease in the number of apoptotic cells (Figure 6B) in both HNE2 and CNE2 cells. DNA damage pathway was partly blocked by inhibited ROS generation *in vitro* and *in vivo* (Figure 6C). These data validate that TMZ-POH induces NPC cell death by activating ROS production.

Previous studies have reported that high endogenous ROS levels correlate with the activation of the JNK pathway and DNA damage response in human cancer cells [33, 34]; ROS-mediated JNK activation–induced DNA damage causes mitochondrial dysfunction-related apoptosis [35]; and ATM, which is well known for its role in the cellular response to DNA breaks, also regulates many diseases through JNK [36]. Besides, ATM phosphorylates H_2_AX and ROS induction, which is partly mediated by increasing H_2_AX. Some inducers of cellular stress, UV irradiation and DNA-damaging agents, can increase the transactivation capacity of ATF-2 through the SAPK/JNK. Phosphorylation of ATF-2 could activate a large set of genes associated with tumorigenesis, maintenance, and physiological homeostasis as well as transcription factors and proteins engaged in stress and DNA damage response, including tumor necrosis factor, transforming growth factor, cyclin A, and cyclin D1 [37].

In addition, the role of JNKs was studied in cell cycle blockade or cell death induced by TMZ-POH to further investigate the mechanisms of these events. JNK activation was directly related to the increased ROS by TMZ-POH, because the blockage of ROS by CAT and NAC blocks JNK phosphorylation in TMZ-POH-treated CNE2 and HNE2 cells. The present report is the first to reveal that TMZ-POH induced an increase in ROS accumulation, and JNK is an essential signaling pathway linking to ROS accumulation in human NPC cells. Taken together, the results indicate that TMZ-POH-induced ROS accumulation is responsible for the upregulation of the JNK pathway, which in turn induces tumor cell death.

The cytotoxicity induced by TMZ-POH was quite different from that by TMZ. TMZ methylates the *N*^7^ and *O*^6^ positions of guanine and the *N*^3^ position of adenine [38], and the methyl adducts, *O*^6^-methylguanine, *N*^7^-methylguanine, and *N*^3^-methyladenine, result in a continuous cycle of DNA base mismatch repair with eventual strand breaks, ultimately leading to cellular apoptosis [39, 40]. Resistance to TMZ emerges with prolonged treatment, mainly due to MGMT, which repairs *O*^6^-methylguanine lesion by transferring the alkyl group from guanine to a cysteine residue, which poses a major therapeutic challenge [41]. The present study supported that the DNA damage induced by TMZ-POH resulted from ROS accumulation, which was quite different from that by TMZ. On the basis of its limited toxicity and ease of administration, TMZ-POH may be used as a long-term maintenance therapy. Such a treatment would prolong survival of patients at a reasonably high level of quality of life.

In summary, the present study showed that TMZ-POH exhibited its cytotoxicity via ROS accumulation, which might result from MMP collapse and lead to activated MAPKs signaling, DNA damage, and cell cycle arrest, and thus inhibit tumor proliferation. It is, therefore, proposed that TMZ-POH should be investigated further as a potentially effective therapy for NPC.

## MATERIALS AND METHODS

### Pharmacological agents

TMZ was purchased from Sigma-Aldrich (China), which were dissolved in DMSO (Sigma-Aldrich) to a concentration of 100 mM. POH and TMZ-POH were provided by NeOnc Technologies Inc. (Los Angeles, CA, USA) and diluted with DMSO to make stock solutions of 100 mM. In all cases of cell treatment, the final DMSO concentration in the culture medium never exceeded 0.5%. Stock solutions of all drugs were stored at −20°C. ROS scavengers, CAT and NAC, were purchased from Sigma-Aldrich.

### Cell culture and treatment

Human NPC cell lines CNE1, CNE2, HNE2, and SUME-α were purchased from the China Center for Type Culture Collection (China) and were cultured in DMEM (Gibco, Invitrogen, CA, USA) and supplemented with 10% FCS (Gibco, Invitrogen) and antibiotics (penicillin/streptomycin, 100 U/mL) at 37°C in 5% CO_2_. Cells were plated in cell culture plates and allowed to adhere overnight, subsequently treated with control (DMSO), TMZ, POH, TMZ plus POH, or TMZ-POH. In some experiments, ROS scavengers, CAT and NAC, were employed 2 hours before the aforementioned treatments.

### Cell viability assay

The effect of TMZ-POH was also evaluated by a conventional MTT cell viability assay, and results were presented as a percentage of the control. Briefly, NPC cells were seeded in triplicates in 96-well plates and treated with various concentrations (0, 40, 80, 120, 160, and 200 μM) of TMZ, POH, TMZ plus POH, and TMZ-POH for 48 hours. Thereafter, 10 μL of the MTT [5 mg/mL in phosphate-buffered saline (PBS), Sigma-Aldrich] stock solution was added, followed by incubation at 37°C in 5% CO_2_ for 24 hours. Formazan crystals that form were solubilized with 100 μL of acidified (0.01 M HCl) 10% sodium dodecyl sulfate (SDS) overnight at 37°C. Absorbance at 570 nm was read on a Bio-Rad 680 microplate reader (Bio-Rad Laboratories, CA, USA), and results were reported relative to a reference wavelength of 630 nm.

### Colony formation assay

Depending on the cell line, 150–350 cells were seeded into each well of a 6-well plate and exposed to the aforementioned treatments. The appropriate plating density was aimed to produce 20–100 surviving colonies per well. These cells were incubated at 37°C for 10–14 days. After fixation with acetic acid–methanol (1:4) and staining with diluted crystal violet (1:30), colonies consisting of > 50 cells were considered and calculated. Results from the triplicate plates were averaged and divided by initial seeded cells to yield the survival rate of clones for each concentration, and the surviving fraction was determined. All survival curves represented a minimum of three independent experiments.

### Cell cycle analysis

Cells were treated by above agents, collected and washed in PBS, then re-suspended and fixed in 70% ethanol overnight. After incubation in 1 ml of propidium iodide staining solution (0.1% Triton X-100, 200 μg/ml DNase-free RNase A, 20 μg/ml propidium iodide) for 1 hour at room temperature, DNA content was evaluated by a FACS Calibur instrument (Becton Dickinson, Bedford, MA, USA) and the distribution of cell cycle phases was determined using ModiFit software (Topsham, ME, USA). Two independent experiments were carried out.

### Detection of apoptotic cells

Apoptosis was evaluated using the Annexin V–FITC Apoptosis Detection Kit (BD Biosciences Pharmingen, CA, USA). Cells were resuspended and incubated with FITC–Annexin V/PI for 15 minutes in the dark, and then evaluated by flow cytometry (FACS Calibur, BD Biosciences) using the Cellquest software.

### *In vivo* studies

All animal protocols were approved by the Institutional Animal Care and Use Committee of Shandong Cancer Hospital and Institute, China. A subline of HNE2 cells called HNE2/luc was used, which was transfected with the firefly luciferase gene. BALB/c-nu mice (4–6 weeks of age, female, from Beijing HFK Bioscience Co., Ltd., China) were subcutaneously injected into the flank with 3 × 10^6^ HNE2/luc cells in 100 μL PBS. The mice were housed in laminar flow cabinets under specific pathogen-free conditions. Seven days after the implantation, efficient tumor volume was confirmed in all animals via noninvasive whole-body bioluminescent imaging. For this purpose, mice were intraperitoneally injected with 50 mg/kg D-luciferin (Perkin Elmer, MA, USA) and imaged using the Xenogen IVIS Spectrum Imaging System (Caliper/Perkin Elmer). Then mice were randomly divided into five groups, five in each group, and treated once a day for 5 days with the following: Control (DMSO), TMZ, POH, TMZ plus POH, and TMZ-POH. Mice were imaged twice per week. Images were analyzed by region-of-interest (ROI) analysis using the Living Image software package (Caliper/Perkin Elmer, MA, USA) to quantitate the tumor volume. Mice were sacrificed and examined for the growth of tumors 17 days after the cessation of drug treatment.

### Determination of ROS production

The production of ROS in cells following the aforementioned treatment was evaluated using the 2′,7′-dichlorofluorescein diacetate (DCFH-DA) kit (Beyotime, China), according to the manufacturer's protocol. Briefly, cells were washed with serum-free DMEM twice and treated with 20 μM DCFH-DA for 30 minutes at 37°C, washed and suspended in PBS. Then the florescence intensity was evaluated by the FACS Calibur instrument.

### Analysis of mitochondrial transmembrane potential

After the aforementioned treatment, cells were stained with the cationic dye 5,5′, 6,6′-tetrachloro1,1′, 3,3′-tetraethyl-benzimidazolylcarbocyanine iodide (JC-1; Beyotime) to demonstrate the state of mitochondrial transmembrane potential (MTP), according to the manufacturer's protocol. Briefly, cells were harvested and transferred to 1.5-mL tubes, and then incubated with JC-1 (5 μg/mL) in a 37°C incubator for 20 minutes after washing twice with PBS. Subsequently, cells were collected and subjected to flow cytometry to detect the change of JC-1 florescence.

### Western blots

Cells were lysed in cell lysis buffer (Beyotime), and the total cellular protein concentration was determined with a BCA Protein Assay Kit (Thermo Fisher Scientific Inc., IL, USA). Equal amounts of protein was separated on sodium dodecyl sulfate–polyacrylamide gel electrophoresis (SDS-PAGE) and transferred onto polyvinylidene fluoride (PVDF) membranes (Millipore, MA, USA). These membranes were probed overnight at 4°C with the following primary antibodies: antibodies against human PARP, p-ATM, p-ATR, γ-H_2_AX, p-Chk1/2, Cyto *c*, p-JNK, ATF-2, *c*-Jun, MKK-4, SDHA, Casp 3, and cleaved Casp 7 (all 1:1000; Cell Signaling Technology, MA, USA), antibody against ACTB (1:2000; Zsbio, China), followed by secondary antibody (Zsbio) with peroxidase for 1 hour at room temperature.

### Enzyme-linked immunosorbent assay

The caspase-3 activity in tissue homogenates was detected by the enzyme-linked immunosorbent assay (ELISA) using a Colorimetric Caspase-3 Assay Kit (Sigma, MO, USA), according to the manufacturer's instructions. Briefly, the synthetic caspase-3 substrate acetyl-Asp-Glu-Val-Asp-p-nitroanilide was added to the reaction mixture, with a control reaction prepared in parallel to exclude any nonspecific hydrolysis of the substrate. Both mixtures were incubated at 37°C for 1–2 hours and the absorbance was read at 405 nm; the caspase-3 activity was expressed as an optical density value at 405 nm.

### Statistical analysis

Statistical significance was evaluated with data from at least three independent experiments. The GraphPad Prism 6.02 software (GraphPad Software, CA, USA) was used for data analysis. All statistical analyses between the two groups were performed using the nonparametric Mann–Whitney test. However, when more than two groups were evaluated, data were assessed by analysis of variance followed by Bonferroni-adjusted post hoc *t* tests for multiple pairs of interest without a priori selection. Data are presented as the mean ± standard deviation (SD). All statistical tests were two sided. Differences were considered statistically significant at *P* < 0.05.
